# Trans-bronchial forceps biopsy for COVID-19 related diffuse parenchymal lung abnormalities

**DOI:** 10.1186/s12890-024-03449-0

**Published:** 2024-12-23

**Authors:** Janina Kleymann, Sascha Brückmann, Simona Langner, Dirk Koschel, Martin Kolditz

**Affiliations:** 1 Medical Department I, Division of Pneumology, University Hospital Carl Gustav Carus, TU Dresden, Fetscherstrasse 74, 01307 Dresden, Germany; 2 East German Lung Center Dresden - Coswig, Dresden - Coswig, Germany; 3 Institute of Pathology and Tumor- and Normal Tissue Bank of the University Cancer Center (UCC), University Hospital Carl Gustav Carus, Medical Faculty, Technische Universität Dresden, Dresden, Germany; 4 Department of Internal Medicine and Pneumology, Fachkrankenhaus Coswig, Coswig, Germany

**Keywords:** Trans-bronchial biopsy, COVID-19 related lung changes, Organizing Pneumonia, Immunocompromised

## Abstract

**Purpose:**

The role of lung biopsy for evaluation of persistent chest radiographic abnormalities including secondary organizing pneumonia (OP) in COVID-19 remains uncertain. This study aimed to evaluate the diagnostic value of trans-bronchial forceps biopsy (TBFB) in patients with persistent lung abnormalities on thoracic computed tomography (CT) scan following SARS-CoV-2 infection with particular focus on cases with OP and immunocompromised (IC) patients.

**Methods:**

Descriptive retrospective single center analysis of all TBFB performed for diffuse lung parenchymal changes after COVID-19 03–2020 to 06–2023.

**Results:**

Twenty seven consecutive TBFB including 23 in IC patients resulted in 100% samples with alveolar tissue showing a high frequency of 12/27 (44%) histological pattern of OP. Steroids were used in 21/27 patients (78%) including 11/12 (92%) with OP. Clinical outcome at discharge was favorable in 89% (92% with OP).

**Conclusion:**

TBFB contributes to the diagnosis of diffuse parenchymal lung abnormalities in the context of COVID-19 including a frequent OP pattern particularly in IC patients. Larger studies are necessary to confirm our data and elucidate on the optimal steroid treatment modality.

**Trial registration:**

Clinical trial number: not applicable. The study was approved by the Ethics Committee of the University Medicine Carl Gustav Carus, TU Dresden (BO-EK-309072023). Waiver of informed consent was granted because of the retrospective nature of the study.

## Background

Patients with COVID-19 can develop a broad spectrum of pulmonary sequelae including persistent interstitial lung changes [1]. Organizing pneumonia (OP) is a frequent histological pattern of COVID-19 related persistent abnormalities on chest computed tomography (CT) scan [2]. A potential underlying mechanism is a virus-mediated epithelial alveolar damage with leakage of plasma proteins and alveolar deposition of fibrin inducing organization into intra-alveolar fibro-inflammatory buds [3]. A trans-bronchial lung biopsy can be helpful in differential diagnosis [4]. While Culebras et al. provided the first results on safety and effectiveness of trans-bronchial cryobiopsy (TBCB) [2], surprisingly little is known about the safety and diagnostic value of trans-bronchial forceps biopsy (TBFB) in COVID-19 associated lung abnormalities.

An empiric systemic steroid trial is recommended for treatment of symptomatic parenchymal lung abnormalities after SARS-CoV-2 infection [5, 6] without consensus on dose and regime. To our best knowledge there is no data in immunocompromised (IC) patients and histologically confirmed OP cases.

Therefore, our study aimed to (1) evaluate safety and diagnostic yield of TBFB in patients with interstitial lung abnormalities after COVID-19 and (2) assess the outcome, especially in the subgroups of patients with histologically confirmed organizing pneumonia and in IC patients.

## Methods

For the current retrospective cohort study, we selected cases with a hospital discharge diagnosis of COVID-19 without need for ventilator support (at the time of lung biopsy) who underwent a flexible bronchoscopy with TBFB in the Respiratory Medicine Department of University Hospital Dresden between March 2020 and June 2023 regardless the reason for hospitalization or invasive procedure (*n* = 48). The pre-selection was based on an International Statistical Classification of Diseases and Related Health Problems (ICD) hospital discharge diagnosis of COVID-19 and the Operation and Procedure classification System (OPS) code for flexible bronchoscopy with lung biopsy. All cases were subsequently clinically validated. The clinical indication for the lung biopsy was determined by the attending respiratory physician.

**Fig. 1:**
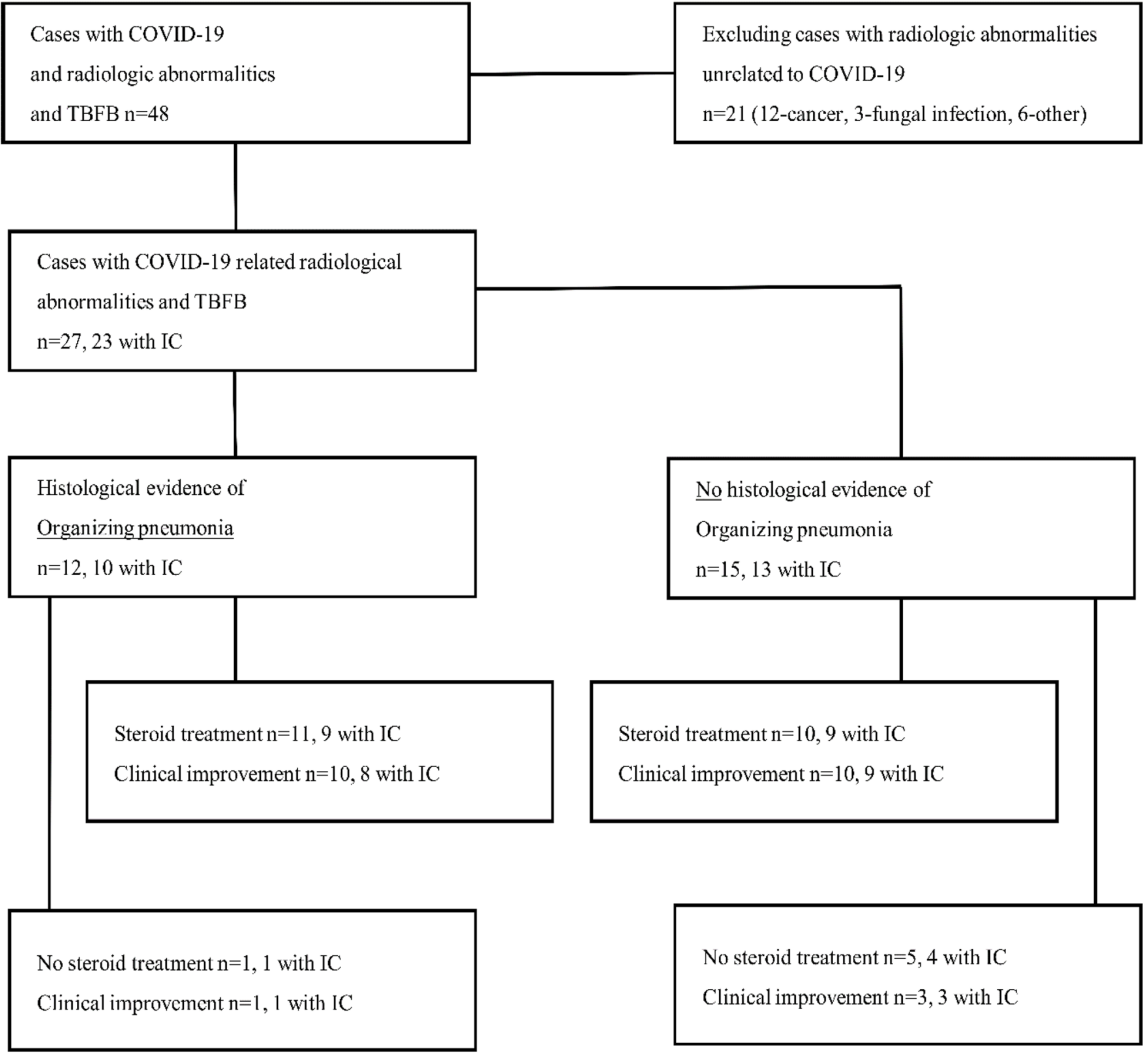
Flow chart on case selection and distribution of patient’s subgroups

Inclusion criteria were (1) PCR confirmed SARS-CoV-2-infection during hospitalization or up to 8 weeks previously, (2) evidence of new COVID-19 related diffuse parenchymal lung radiologic abnormalities demonstrated by chest high-resolution computed tomography in accordance with the recommendations of the Fleischner Society, (3) flexible bronchoscopy with TBFB, performed at our department. Patients with any form of ventilator support at the time of lung biopsy and with radiographic changes not related to COVID-19 (such as tumor or metastatic masses) were excluded (Fig. 1).

Demographic characteristics, clinical and histological data, comorbidities, particularly IC conditions, modality of systemic steroid treatment and outcome were retrieved from the electronic patient data file. The severity of COVID-19 was classified according to WHO Clinical Progression Scale [7]. The Charlson Comorbidity Index (CCI) was used to quantify patients' comorbidities [8]. The severity of dyspnea was assessed by Modified Medical Research Council Dyspnea Scale (mMRC).

Patients were qualified as IC according to JA Ramirez et al. [9] if they had a primary immune deficiency disease, active solid or hematologic malignancy, HIV infection with a CD4 T-lymphocyte count < 200 cells/mL or < 14%, solid organ/ hematopoietic stem cell transplantation, were receiving cancer chemotherapy or ≥ 20 mg prednisone equivalent dose per day with cumulative dose > than 600 mg or biological immune modulators/disease-modifying antirheumatic drugs/other immunosuppressive drugs within the last 3 months.

The patients` clinical condition was assessed daily during their hospital stay. Discharge was planned once sustained clinical improvement was achieved. Criteria of clinical improvement were: heart rate < 100/min, breathing rate < 24/min, systolic blood pressure > 90 mmHg, body temperature < 37,8°C, oral food intake ability, pO2 > 60 mmHg or SaO2 > 90% and intact consciousness [10]. Positive treatment outcome was defined as patients meeting all the following criteria at hospital discharge: (1) documented clinical improvement, (2) declining oxygen requirements and (3) decreasing C-reactive protein.

Descriptive data analysis was performed. The collected results are presented as median with the corresponding interquartile range (IQR).

## Results

### Patient characteristics

A total number of 48 cases with COVID-19 and interstitial changes on CT scan who had TBFB performed in our department were screened. 21 cases were excluded due to lack of evidence of COVID-19 associated diffuse parenchymal lung abnormalities. 27 cases met eligibility criteria, including 23 IC patients. The detailed description of the case selection is depicted in Fig. 1. Table 1 shows main characteristics of the included patients.

**Table 1:** Patients` characteristics, histological pattern, treatment and outcome

	Overall (*N* = 27)	OP (*N* = 12)	non-OP (*N* = 15)	IC (*N* = 23)	non-IC (*n* = 4)
***Demography***					
Age (years), median (IQR)	56 (25.5)	58 (16.5)	53 (34)	54 (28.5)	74 (6.25)
Male, n (%)	16 (60)	7 (58)	9 (60)	14 (61)	2 (50)
***Severity of COVID-19***					
WHO severity scale, median (IQR)	4 (1)	4 (1)	4 (1)	4 (1)	4.5 (1)
***Comorbidities***					
IC, n (%)	23 (85)	10 (83)	13 (87)		
Active malignancy total/solid/ hematologic, n (%)	15 (56)	8 (67)/2(17)/6(50)	7 (47)/1(7)/6(40)		
Solid organ transplantation, n (%)	6 (22)	2 (17)	4 (27)		
HSCT, n (%)	2 (7)	1 (8)	1 (7)		
Receiving cancer chemotherapy, n (%)^a^	6 (22)	3 (25)	3 (20)		
Receiving ≥ 20 mg prednisolone/d	1 (4)	1 (8)	0 (0)		
Other immunosuppressive treatments, n (%)^b^	6 (22)	3 (25)	3 (20)		
CCI, median (IQR)	4 (3)	4 (2.75)	3 (6)	4 (3)	5 (3.75)
***Clinical parameters at time of bronchoscopy***					
FVC %, median (IQR)	81.5 (20)	80 (22.3)	80.5 (22)	79 (25)	85 (2)
DLCO %, median (IQR)	55 (16)	57 (21)	54 (17)	54 (15.3)	55 (11.5)
mMRC, median (IQR)	0.5 (2)	0.5 (2.3)	0.5 (2)	1 (2)	0 (0.3)
O2 supplementation, n (%)	10 (37)	4 (33)	6 (40)	8 (35)	2 (50)
O2 rate (l/min), median (IQR)	2.5 (2)	4.5 (3)	2 (2)	3 (2)	2 (0)
CRP mg/l, median (IQR)	99 (88)	124 (113)	49.5 (86.5)	69.5 (82)	121.5 (52.5)
***CT Scan***					
Ground-glass opacity, n (%)	19 (70)	8 (67)	11 (73)	17 (74)	2 (50)
Consolidation, n (%)	10 (37)	5 (42)	5 (33)	8 (35)	2 (50)
Reticulations, n (%)	2 (7)	0 (0)	2 (13)	1 (4)	1 (25)
***Histological pattern of TBFB***					
Organizing pneumonia, n (%)	12 (44)			10 (43)	2 (50)
Inflammation, n (%)	8 (30)				
Histiocytic reaction, n (%)	2 (7)				
Normal or subtile nonspecific findings, n (%)	2 (7)				
Diffuse alveolar damage, n (%)	1 (4)				
Fibrosis, pattern not definable, n (%)	1 (4)				
Alveolar proteinosis, n (%)	1 (4)				
***Treatment***					
Steroids, n (%)	21 (78)	11 (92)	10 (67)	18 (78)	3 (75)
Initial Prednisolone dose mg/d, median (IQR)	60 (59)	60 (43)	50 (60)	60 (56.5)	55 (70)
***Outcome***					
Peri-interventional complications, n (%)	2 (7)	1 (8)	1 (7)	2 (9)	0 (0)
Hospital mortality, n (%)	2 (7)	1 (8)	1 (7)	1 (4)	1 (25)
Clinical improvement at discharge, n (%)	24 (89)	11 (92)	13 (87)	21 (91)	3 (75)

*CCI* Charlson Comorbidity Index, *CRP* C-reactive protein, *CT* Computed Tomography, *d* day, *DLCO* diffusing capacity of the lungs for carbon monoxide, *IC* immunocompromised, *IQR* interquartile range, *FVC* forced vital capacity, *HL* Hodgkin Lymphoma, *HSCT* Hematopoietic stem-cell transplantation, *mMRC* modified Medical Research Council Dyspnea Scale, *NHL* Non-Hodgkin Lymphoma, *O2* oxygen, *WHO* World Health Organization

^a^The following chemotherapy regimens were used: Carboplatin/Etoposide in 1 patient with lung cancer, Bendamustine in 1 patient with NHL, BEACOPP in 1 patient with HL, combination chemotherapy (unknown regimen) in 3 patients with NHL

^b^The following other immunosuppressive treatments were used: Obinutuzumab (2 cases in OP and 1 in non-OP group), Ocrelizumab in 1 case from non-OP group, Ruxolitinib in 1 case from non-OP group, Teclistamab in 1 case from OP group

Detailed description of the causes of IC are provided within Table 1. No cases with primary immune deficiency diseases, HIV infection or patients on any disease-modifying antirheumatic drugs were present in our study population.

Microbiological analysis of the bronchoalveolar lavage (BAL) fluid revealed an additional bacterial isolate in 7 from 11 OP patients (3 samples with Staphylococcus aureus, 1 Serratia marcescens, 1 Stenotrophomonas maltophilia, 1 Enterobacter cloacae and 1 Escherichia coli) and in 4 from 15 non-OP patients (2 Staphylococcus aureus, 1 Klebsiella oxytoca, 1 Klebsiella pneumonia). The median percentage of lymphocytes present in the bronchoalveolar lavage fluid was 5% (IQR 19).

### Computed tomography scan of the chest

**Fig. 2:**
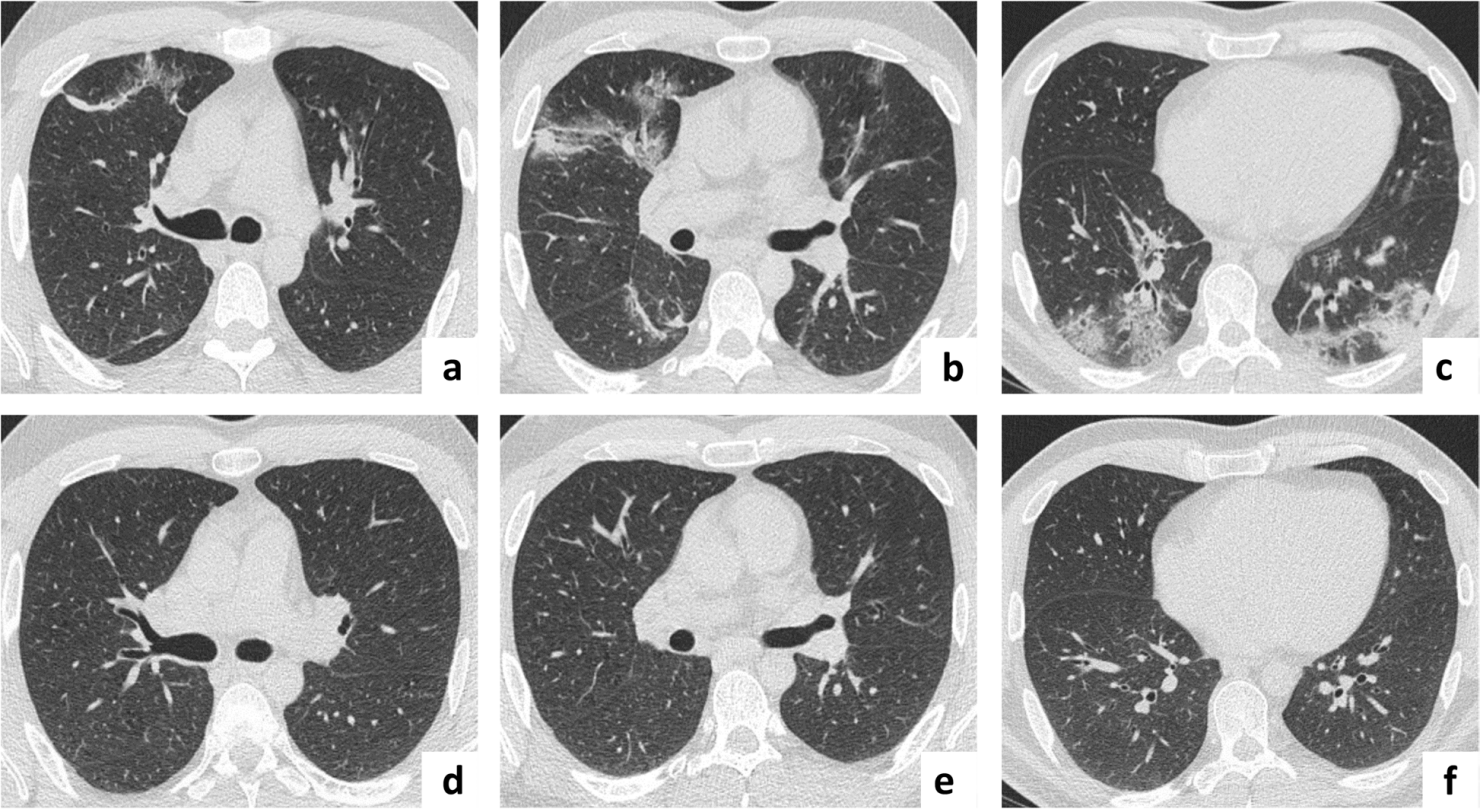
COVID-19 associated radiological changes on CT scans before and after steroid treatment. Thoracic CT scan from a 38–year-old male COVID-19 patient with histologically proven OP showing bilateral ground glass opacities and multifocal consolidations with a peripheral lung and subpleural distribution in the (**a**) upper, (**b**) middle and (**c**) lower field during acute infection before steroid treatment. CT scan 4 months later shows near complete resolution of radiological abnormalities in the (**d**) upper, (**e**) middle and (**f**) lower field after steroid treatment

Before treatment initiation 19 patients (70%) had Ground-glass opacities, 10 cases (37%) consolidations and 2 (7%) reticulations on the CT imaging. Figure 2 shows an example of radiological abnormalities before and after steroid treatment in a patient with histologically proven OP.

### Histopathology of lung biopsy

**Fig. 3:**
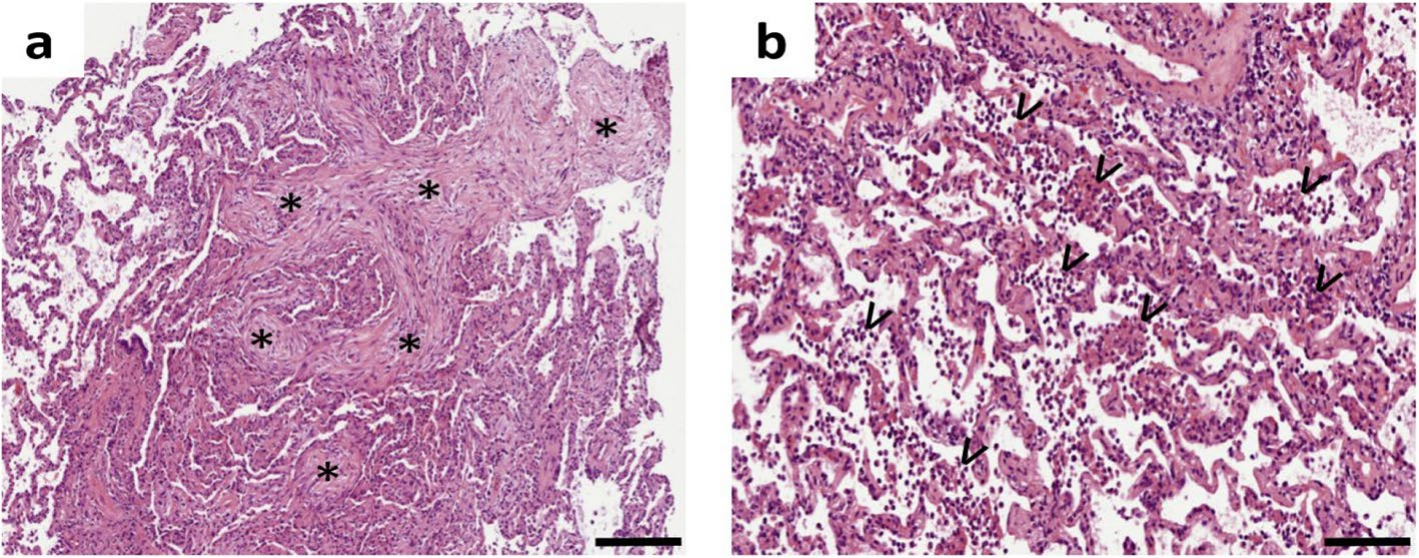
Two representative forceps biopsied specimens from different patients. Hematoxylin and eosin staining, a scale bar in the lower right corner indicates 100 µm. **a** A case with histological pattern organizing pneumonia: Slides revealing intra-alveolar buds of granulation tissue (Masson Bodies, asterisk), loose connective matrix extending into the lumen of distal bronchioles. **b** A patient with morphological evidence of inflammation: sections showing moderate intra-alveolar inflammation (arrowheads)

All 27 samples showed alveolar tissue. An OP pattern was histologically confirmed in 12 cases (44%), including 10 IC cases (43% of IC). 8 samples (30%) revealed inflammatory changes: 1 granulocytic, 1 lymphogranulocytic and 6 minimal nonspecific inflammation. Further histopathologic findings are presented in Table 1. Figure 3 shows two representative histopathologic examples.

### Peri-interventional complications

Complications were observed in 2 cases, both patients were IC. One patient with OP and a history of lung transplantation for chronic hypersensitivity pneumonia and myelodysplastic syndrome developed short-term bleeding which was cupped by endobronchial instillation of Tranexamic Acid. One patient without OP after kidney transplant and background of systemic scleroderma and suspected pulmonary hypertension had a temporary peri-interventional desaturation and bleeding which was managed by local application of 1% Xylometazoline. None required a ventilator support.

### Steroid treatment

Steroid treatment with prednisolone (as the mostly used steroid in Germany) was initiated as indicated by the attending respiratory physician in 21 cases (78%): in 11/12 cases with OP (9/10 with IC and 2/2 without IC) and in 10/15 cases in the non-OP group (9/13 with IC and 1/2 without IC). The median prednisolone starting dose was 60 mg/d (IQR 59) or 1 mg per kilogram of body weight per day (IQR 0.7).

### Outcome

The positive treatment outcome was reached in 24/27 cases overall (11/12 with OP, 13/15 without OP, 21/23 with IC and 1/4 without IC). The median length of hospital stay was 8 days. Among patients treated with steroids, clinical improvement at discharge was achieved in 10/11 OP cases and in 10/10 non-OP cases. In-hospital death was observed in 2/27 patients. Both cases did not have any biopsy-related complications such as post-interventional bleeding or pneumothorax. One patient with pre-existing hemiparesis, percutaneous endoscopic gastrostomy, tracheostomy and nonspecific lung changes on biopsy died suddenly 3 days after bronchoscopy and had no signs of respiratory deterioration and was not on any steroid treatment. A Do Not Resuscitate order was agreed in advance. The second patient had a pre-existing IPF, a history of small cell lung cancer (chemotherapy completed 9 months ago) and surgically treated non-small cell lung cancer (lower lobe resected 3 years ago). This patient`s lung biopsy sample revealed an OP pattern, so steroid treatment was initiated. The patient died from respiratory failure despite oxygen treatment via facemask with 15 l/min 3 days after the bronchoscopic procedure. There was no change in clinical dynamics over time. Any form of mechanical ventilation was refused in advance. None of the fatalities were associated with the bronchoscopic procedure.

## Discussion

This study assessed the value of TBFB in diffuse lung parenchymal changes after COVID-19. The main findings of our study can be summarized as 1) a histologic OP pattern is frequently demonstrated by TBFB in the context of COVID-19 associated diffuse parenchymal lung abnormalities, 2) steroids were used in most patients regardless of histological confirmation of OP but in nearly all patients with OP and 3) the clinical outcomes in OP and non-OP subgroups were broadly similar.

An OP pattern was the most frequent histopathological pattern in the overall group (*n* = 12, 44%), in the subgroup of IC (*n* = 10, 43%) and non-IC (*n* = 2, 50%). However, other histologic patterns with potential treatment implications as depicted in Table 1 were present. Similar frequencies of OP have been reported by other studies using TBCB: *n* = 16, 32% [2], *n* = 5, 33% [11], and *n* = 4, 40% [12]. While we assessed hospitalized patients with SARS-CoV-2 infection up to 8 weeks beforehand, the study of Culebra [2] included only patients referred to post-COVID clinic after hospitalization with a negative SARS-CoV-2 nasopharyngeal swab. 16 of 49 representative samples in their study had signs of OP. The study of Ravaglia et al. [12] provided the histological data on 10 TBCB from patients with persistent lung involvement on CT scan and respiratory or systemic symptoms without resting hypoxemia. All patients had negative results on SARS-CoV-2 molecular swab testing (average 3.5 months after recovery). TBCB revealed a pattern consistent with OP in 3 of 10 cases. Another study from Doglioni et al. [11] reported immunohistochemical and morphological features detected in 15 TBCB samples from COVID-19 patients with diffuse parenchymal lung changes in early stages of COVID-19 pneumonia (within 20 days of symptom onset). In contrast to our predominantly multimorbid cohort, this study included patients with only few or no comorbidities. Analogous to our data, 3 samples had a pattern of OP and 2 had mixed pattern of OP with non-specific interstitial pneumonia.

Most patients (78%) in our study were treated with steroids. However, fewer patients without histological OP (67%) were treated with steroids than with confirmed OP (92%), thus histological evidence might have influenced treatment modality. Given the evidence of clinical, lung functional and CT scan improvement [6], steroids are recommended for symptomatic COVID-19 related OP analogous to other secondary OP [4], though critical evaluation due to steroid side effects is warranted. Therefore, in our study TBFB might have been one decisive factor for reducing the number of steroid treated patients by 25%. This is comparable to the observed reduction of steroid use by 34% after TBCB obtained histology for COVID-19 related lung abnormalities in [2].

The exact dose and duration of corticosteroids administration in COVID-19 related OP is unknown. The median starting dose of prednisolone in our cohort was 60 mg (IQR 59) daily. It was slightly higher in the OP group at 60 mg versus 50 mg in the non-OP group. These doses are comparable with ones recommended for secondary OP forms but are higher than reported doses for COVID-19 related OP by others: An open-label randomized trial on post-COVID-19 parenchymal lung abnormalities on CT chest reported that a higher prednisolone dose of 40 mg/d may not be superior to 10 mg/d [5]. A non-randomized observational study showed marked clinical improvement of symptomatic patients on an average starting dose of 26 mg/d of prednisolone in clinically suspected OP after COVID-19 [6]. No histological examination was carried out in both studies. There is no data on the optimal steroid dose in histologically proven post COVID-19 OP.

The clinical improvement at discharge was consistently high with 89% overall and 92% in OP similar to published data [5, 6].

The rate of peri-interventional complications was low in our study (*n* = 2, 7%) and included two patients with moderate bleedings which were controlled locally (application of vasoactive substances). In contrast to our results the frequency of moderate hemorrhage was much higher (40%, *n* = 20) in the study of Culebras [2], who used TBCB in patients with COVID-19 suggestive pulmonary sequelae after hospitalization for COVID-19. Two other recent studies on TBCB for parenchymal lung abnormalities after COVID-19 did not analyze peri-interventional complications [11, 12].

Our study population had a remarkably high proportion of 85% of immunocompromised patients. The proportion of IC patients in other studies investigating persistent lung abnormalities after COVID-19 was far below with 8% in [2] and 3% in [6]. Therefore, our data demonstrating OP as most frequent histological pattern and clinical response to steroids in most IC patients are of particular relevance for this rarely investigated patient group.

Most of our IC patients had an iatrogenic nature of immunosuppression (immunosuppressive treatment after transplants, chemotherapy). Given that OP pattern can also be induced by chemo/immunotherapy [13–15], we cannot exclude a potential association with treatment. However, given the direct temporal association with the positive SARS-CoV-2 PCR and the main discharge diagnosis of COVID-19 this seems less likely.

Major limitations of our study include the retrospective design, small sample size, lack of a standardized steroid treatment protocol and absence of any structured post-discharge follow-up. A standardized steroid treatment protocol is urgently needed to determine the appropriate starting dose and tapering modality. A structured post-discharge follow-up of the clinical, functional and radiological findings, taking comorbidities into account should be evaluated in future studies. Additionally, we cannot exclude the possibility that some of the histologic findings including OP were related to underlying diseases or treatments rather than the SARS-CoV-2 infection.

## Conclusion

TBFB for diffuse parenchymal lung abnormalities in the context of COVID-19 frequently detected a histologic pattern of OP particularly in patients with IC and thus might add to the diagnostic workup. Most cases were treated with prednisolone with favorable treatment effect. Larger studies are necessary to confirm our data and elucidate on the optimal steroid treatment modality.

## Data Availability

The datasets used and analysed during the current study are available from the corresponding author on reasonable request.

## Abbreviations

BAL: Bronchoalveolar lavage
CCI: Charlson Comorbidity Index
CT: Computed tomography
HL: Hodgkin Lymphoma
IC: Immunocompromised
ICD: International Statistical Classification of Diseases and Related Health Problems
IQR: Interquartile range
mMRC: Modified Medical Research Council Dyspnea Scale
NHL: Non-Hodgkin Lymphoma
O2: Oxygen
OP: Organizing pneumonia
OPS: Operation and Procedure classification System
TBCB: Trans-bronchial cryobiopsy
TBFB: Transbronchial forceps biopsy
WHO: World Health Organisation
